# BC-Box Motif in SOCS6 Induces Differentiation of Epidermal Stem Cells into GABAnergic Neurons

**DOI:** 10.3390/ijms21144947

**Published:** 2020-07-13

**Authors:** Tetsuya Yoshizumi, Atsuhiko Kubo, Hidetoshi Murata, Masamichi Shinonaga, Hiroshi Kanno

**Affiliations:** 1 Department of Neurosurgery, International University of Health and Welfare Atami Hospital, Atami 413-0012, Japan; tyoshizumi@iuhw.ac.jp (T.Y.); macshino@iuhw.ac.jp (M.S.); 2 Nerve Care Clinic, Yokosuka 238-0012, Japan; dr.kubo@nccncs.com; 3 Department of Neurosurgery, Yokohama City University Graduate School of Medicine, Yokohama 236-0004, Japan; hmurata@yokohama-cu.ac.jp

**Keywords:** SOCS6 peptide, epidermal stem cells, GABAnergic neuron, neuronal differentiation

## Abstract

The BC-box motif in suppressor of cytokine signaling 6 (SOCS6) promotes the neuronal differentiation of somatic stem cells, including epidermal stem cells. SOCS6 protein belongs to the group of SOCS proteins and inhibits cytokine signaling. Here we showed that epidermal stem cells were induced to differentiate into GABAnergic neurons by the intracellular delivery of a peptide composed of the amino-acid sequences encoded by the BC-box motif in SOCS6 protein. The BC-box motif (SLQYLCRFVI) in SOCS6 corresponded to the binding site of elongin BC. GABAnergic differentiation mediated by the BC-box motif in SOCS6 protein was caused by ubiquitination of JAK2 and inhibition of the JAK2-STAT3 pathway. Furthermore, GABAnergic neuron-like cells generated from epidermal stem cells were transplanted into the brain of a rodent ischemic model. Then, we demonstrated that these transplanted cells were GAD positive and that the cognitive function of the ischemic model rodents with the transplanted cells was improved. This study could contribute to not only elucidating the mechanism of GABAnergic neuronal differentiation but also to neuronal regenerative medicine utilizing GABAnergic neurons.

## 1. Introduction

The suppressor of cytokine signaling (SOCS) proteins act as negative feedback inhibitors. The discovery of the SOCS family of proteins began in 1995, when a novel cytokine-inducible SH2-containing protein (CIS) was isolated as an immediate early gene induced by IL-2, IL-3, and erythropoietin (Epo) [1]. Then, an SH2 domain-containing protein that inhibited interleukin-6 (IL-6)-induced macrophage differentiation was identified in 1997 and designated as suppressor of cytokine signaling 1 (SOCS1) [2,3,4]. The database searches using the predicted amino acid sequence of SOCS1 revealed that at least 20 proteins in mice and humans share sequence homology within a 40-residue C-terminal motif referred to as the SOCS box. The SOCS box-containing proteins were subclassified into subfamilies based on the domains contained in their central regions, and the family member proteins containing a central SH2 domain were termed SOCS (CIS and SOCS1–SOCS7). Each SOCS protein contains 3 distinct domains: an N-terminal domain of low conservation, a conserved central Src-homology 2 (SH2) domain, and a more highly conserved C-terminal domain termed the SOCS box. The N-terminal domain is variable in length among members, SOCS1–3 and CIS having a shorter N-terminal domain in comparison to SOCS4–7 [5]. The SOCS box motif recruits an E3 ubiquitin ligase complex consisting of Elongins B and C, Rbx2, and Cullin5. This complex forms an E3 ubiquitin ligase which tags target proteins such as the Janus kinases (JAKs) and cytokine receptors with ubiquitin, marking them for proteasomal degradation [6,7]. SOCS proteins can regulate signal transduction by linking their substrates to the ubiquitination machinery via the SOCS box. Ubiquitination of a substrate via SOCS proteins can lead to its proteasomal degradation. The expression of SOCS proteins can be induced by cytokine stimulation, and they serve not only to interfere with signaling from the inducing cytokine in a negative feedback loop but also to regulate signaling downstream of other cytokines, a process known as “cross-talk” [8,9]. So far numerous different molecules such as interferons, a large number of cytokines, growth factors, and hormonal factors are known to activate the JAK-STAT pathway, which is inhibited by SOCS proteins.

SOCS proteins are associated with the differentiation of various cell types. As for differentiation of T cells, SOCS1 in T helper (Th) cells is induced by IL-6 and promotes Th17 differentiation [10]. On the other hand, SOCS3 promotes bone marrow cells to differentiate into CD8+ T lymphocytes in lung tissue via the up-regulation of Notch1 [11]. SOCS proteins also play important roles in neuronal differentiation, neurogenesis, nervous system development, and nerve regeneration. Among the members of this family, SOCS1, SOCS2, and SOCS3 are expressed in the nervous system throughout development [12]. SOCS1 regulates the interferon gamma-mediated survival of sensory neurons [13] and regulates the proliferation and neuronal differentiation of neural stem cells (NSCs) [14,15]. SOCS2 is a negative regulator of growth hormone signaling, regulating neurite outgrowth [16,17,18]. Moreover, SOCS2 was also demonstrated to promote neurite outgrowth, regulate neuronal morphology, induce neurogenesis, and inhibit astrogliogenesis of NSCs [16,19]. SOCS3 associates with the IGF receptor and inhibits the JAK/STAT3 pathway [20], and it was also found that SOCS3 induces neuronal differentiation and promotes neural cell survival in PC12 cells [21]. In addition, it was found that SOCS6 also promotes neuronal differentiation of NSCs [22].

SOCS proteins are associated with neuronal diseases such as Alzheimer’s disease (AD), Parkinson’s disease (PD), multiple sclerosis (MS), amyotrophic lateral sclerosis (ALS), ischemic stroke (IS), and traumatic brain injury (TBI) [23]. These diseases are characterized by neuroinflammatory responses including an increased expression of inflammatory cytokines in the brain. SOCS proteins such as SOCS1, 2, 3, 6 play crucial roles in this increased expression of these cytokines [24]. SOCS1 is associated with PD, AD, MS, and IS [24,25,26,27,28], whereas SOCS3 is associated with AD, MS, IS, and TBI [29,30,31,32]. Associations with SOCS2 in AD and SOCS6 in IS have also been reported [25,33,34]. In addition, it has been demonstrated that SOCS1, SOCS2, and SOCS3 are down-regulated in transgenic AD [35]. Although the expression of *SOCS* genes does not change to a large extent in AD brains, there are significantly increased levels of *SOCS2* and *SOCS3* mRNAs and increased levels of SOCS4 and SOCS7 proteins in AD brains [36].

Recently, we demonstrated that peptides containing the BC box motif in some BC-box proteins, such as von Hippel–Lindau (VHL) protein, promote the neuronal differentiation of somatic stem cells [37]. However, the mechanism of neuronal differentiation by BC-box motif in SOCS6 has been never elucidated. Here, we show that the BC-box motif peptide derived from SOCS6 induces GABAnergic neuronal differentiation of epidermal stem cells via inhibition of the JAK2/STAT3 pathway. Furthermore, we propose that GABAnergic neuron-like cells would be promising for neural regeneration following brain ischemia. In addition, we discuss the mechanism of neuronal differentiation via BC-box motif peptide derived from SOCS6.

## 2. Result

### 2.1. Characterization of Pluripotent Epidermal Stem Cells

**Figure 1:**
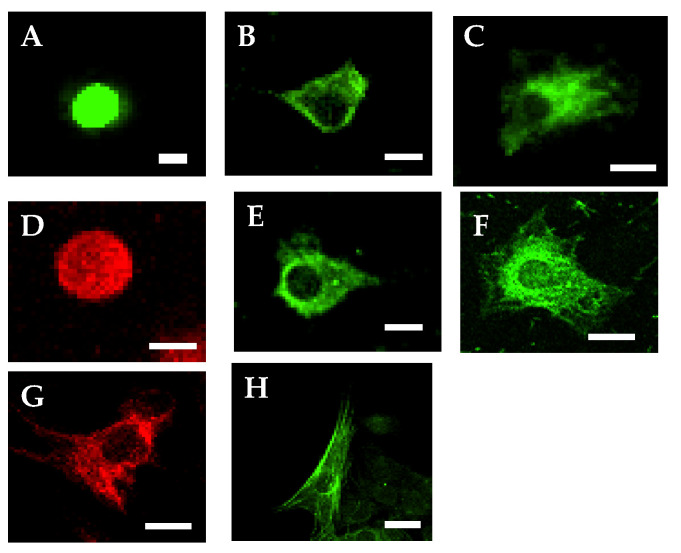
Immunofluorescent images of naïve epidermal stem cells or spontaneously differentiated cells. (**A**), p75NTR stain; (**B**), fibronectin stain; (**C**), nestin stain; (**D**), ret proto-oncogene product (RET) stain; (**E**), Keratin stain; (**F**), glial fibrillary acidic protein (GFAP) stain; (**G**), Neurofilament-M stain; (**H**), Smooth muscle actin stain. Fluorescein isothiocyanate (FITC), green; TRIC, red; Scale bar = 10 μm.

In this study, we used rodent skin-derived precursor cells isolated by the authors as epidermal stem cells [38], the origins of which are pluripotent epidermal neural crest stem cells in the papillae of hair follicles [39]. At first, we characterized the pluripotent epidermal stem cells. After the floating spheres were dissociated, the dissociated cells were cultured for 1 week on cover glasses in wells containing DMEM with 1% FCS, and then an immunocytochemical study was performed. The result showed that 58.3% ± 5.3% of the cells were immunopositive for p75 neurotrophin receptor (NTR, a mesenchymal precursor marker); 78.6% ± 6.6%, for fibronectin (a fibroblast marker); 38.3% ± 3.3%, for nestin (a neural progenitor marker); 71.1% ± 6.4%, for ret proto-oncogene product (RET, an endodermal precursor marker); and 73.4% ± 567%, for keratin (an epidermal stem cell marker). We also found small fractions of cells immunoreactive with antibodies against glial fibrillary acidic protein (GFAP, an astroglial marker, 3.4% ± 0.4%), neurofilament triplet M (NF-M, a neuron marker, 2.1% ± 0.2%), and smooth muscle actin (SMA, a smooth muscle cell marker, 1.4% ± 0.2%; Figure 1). These results suggested the epidermal stem cells to be pluripotent stem cells.

### 2.2. Intracellular Delivery of FITC-Labeled SOCS6 Peptide into Epidermal Stem Cells

**Figure 2:**
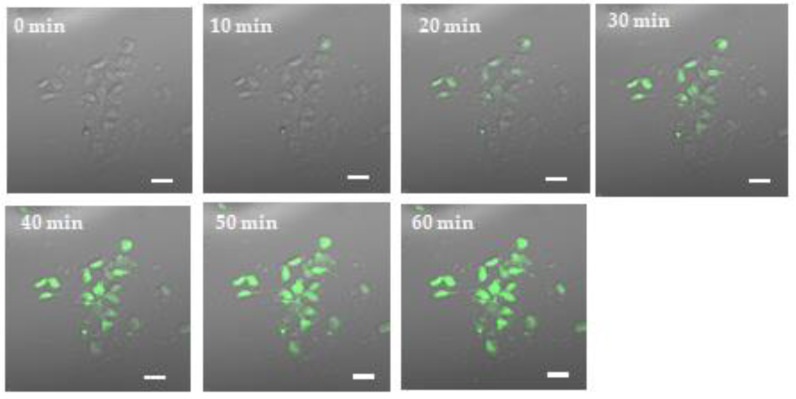
Confocal microphotograph showing internalization of FITC-labeled protein-transduction-domain (PTD)-linked suppressor of cytokine signaling 6 (SOCS6) peptide in the time-lapse imaging of living epidermal stem cells. The internalization of the fluorescent peptide was observed starting at 10 min after the addition of the peptide to the culture medium. By 60 min the peptide was delivered into 77.8% ± 6.7% of the cells. Scale bar = 20 μm.

We synthesized fluorescein isothiocyanate (FITC)-labeled protein-transduction-domain (PTD)-linked BC-box motif peptide derived from SOCS6 (FITC-YARAAARQARASLQYLCRFVIRQYTR). Then, using it we examined the time course of the intracellular delivery of SOCS6 peptide and the rate of intracellular delivery into cultured epidermal stem cells. The time course of subcellularly localized SOCS6 peptide visualized with 2-μM FITC-labeled-PTD-linked peptide in cultures of epidermal stem cells was observed with a confocal microscope. The submembrane translocation of the fluorescent peptide was observed starting 10 min after the addition of the peptide to the culture medium. By 60 min the peptide transduction was visualized in 77.8% ± 6.7% of the cells, and the rate did not increase any further thereafter, suggesting that the intracellular delivery of PTD-linked peptide was completed in 60 min (Figure 2).

### 2.3. SOCS6-Derived Peptide Induce the GABAnergic Differentiation of Epidermal Stem Cells

**Figure 3:**
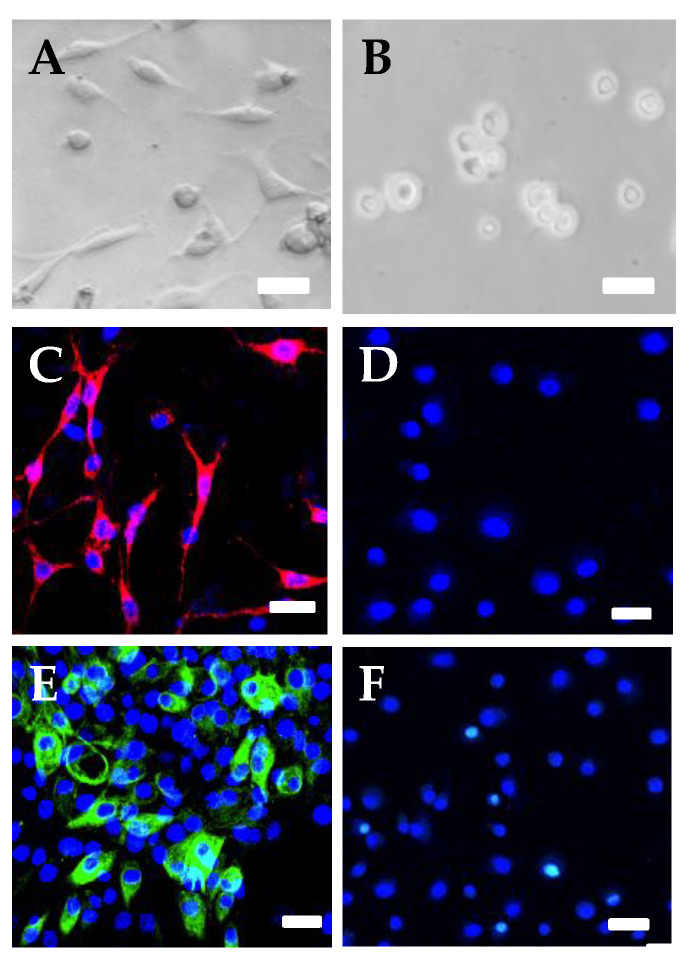
Microphotographs showing cultured SOCS6-peptide-treated epidermal stem cells and non-treated epidermal stem cells. (**A**), The phase-contrast image of SOCS6-treated epidermal stem cells; (**B**), The phase-contrast image of non-treated epidermal stem cells; (**C**), Immunofluorescent image showing SOCS6-treated epidermal stem cells using anti-GAD antibody; (**D**), The immunofluorescent image of non-treated epidermal stem cells using anti-GAD antibody; (**E**), The immunofluorescent image of SOCS6-treated epidermal stem cells using anti-GABA antibody; (**F**), The immunofluorescent image of non-treated epidermal stem cells using anti-GABA antibody TRIC for GAD, red. FITC for GABA, green fluorescence. DAPI for nucleus, blue. Scale bar = 10 μm.

**Figure 4:**
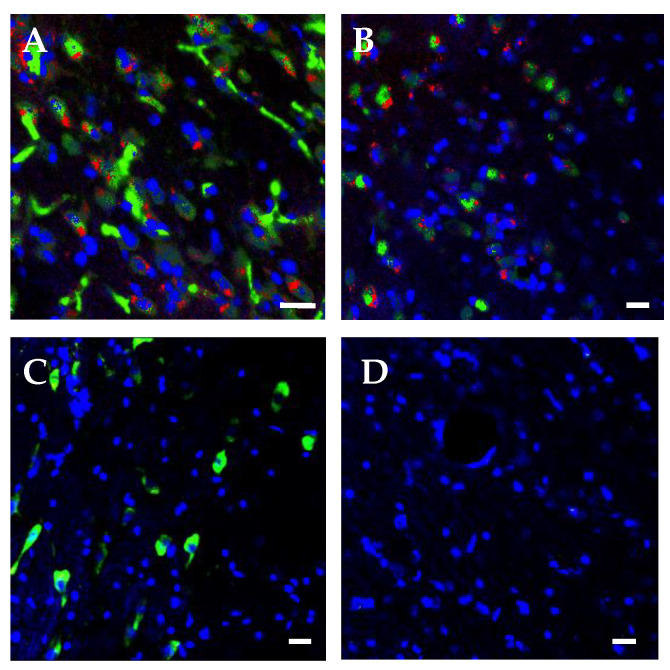
Integration of SOCS6-peptide-treated epidermal stem cells engrafted into the brain of ischemic model rats. Confocal microscope images of engrafted cells with PKH-prelabeling in SOCS6-treated cells (**A**,**C**) and the non-treated cells (**B**,**D**), (**A,B**) Immunohistochemistry using anti-NeuN antibody (FITC, green), PKH (red), and DAPI (blue). PKH-prelabeled cells are more positive for NeuN in engrafted SOCS6-peptide-treated cells (**A**) than non-treated cells (**B**). (**A**–**D**) Immunohistochemistry using anti-GAD antibody (FITC, green) and DAPI (blue). In engrafted SOCS6-peptide-treated cells, GAD-positive cells were found in engrafted SOCS6-peptide-treated cells (**C**), while GAD-positive cells were not found in grafted non-treated cells (**D**). Scale bar = 20 μm.

**Figure 5:**
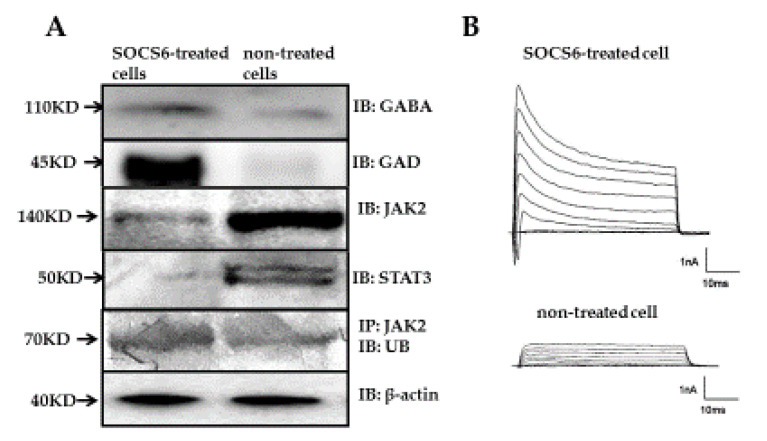
(**A**), Immunoblotting (IB) and immunoprecipitation (IP) study on SOCS6-peptide-treated and non-treated cells. Immunoblotting was done with anti-GAD antibody, anti-GABA antibody, anti-JAK2 antibody, anti-STAT3 antibody, and anti-β-actin antibody (internal control). Immunoprecipitation with anti-JAK2 antibody was followed by immunoblotting with anti-ubiquitin antibody; (**B**), Electrophysiological properties of peptide-treated cells. Voltage-gated inward and outward currents were recorded in the whole-cell patch-clamp configuration. Upper trace, a SOCS6-peptide-terated cell elicited both large outward potassium currents and fast inward Na^+^ currents. Lower trace, a non-treated cell elicited small outward K+ currents and minute fast inward Na^+^ current.

In the morphological analysis, neurite outgrowth > 5 μm was assessed as significance. A proportion of 75.7% ± 7.5% of the SOCS6-peptide-treated cells showed neurite outgrowth (Figure 3A), a very significant difference compared with other cells. The immunocytochemical study on the SOCS6-peptide-treated cells showed the distinct expressions of GABA and GAD (Figure 3B,C). In addition, immunohistochemical analysis revealed that SOCS6 peptide-treated cells differentiated to neuronal marker (NeuN)-positive cells in rat brain (positive rates of NeuN, 42.5% ± 4.5%), whereas non-treated cells differentiated less to NeuN-positive cells (positive rates of NeuN, 9.2% ± 1.8%, *p* < 0.05). In addition, SOCS6-peptide-treated cells showed enhancement of the sprouting. Furthermore, 18.2% ± 3.1% of the SOCS6-peptide-treated cells transplanted into rat brain were GAD positive, whereas non-treated cells were not (Figure 4). Western blotting revealed that the levels of GAD and GABA proteins were markedly increased in the SOCS6-peptide-treated cells (Figure 5A), thus indicating that the SOCS6-peptide-treated cells showed neuronal differentiation to the GABAnergic phenotype. These experiments were performed at least twice. To assess the frequency of different cell types in a given culture, we counted the number of cells immunopositive with a given antibody in 6 random non-overlapping visual fields (200 cells per field) in each experiment.

### 2.4. Inhibition of JAK-STAT Pathway and Neuronal Differentiation

Next, we asked whether inhibition of the JAK-STAT pathway was associated with the neuronal differentiation of the epidermal stem cells achieved via intracellular delivery of the SOCS6 peptide. We first observed by Western blotting analysis using anti-STAT3 and anti-JAK2 antibodies that the protein levels of JAK2 and STAT3 were reduced significantly in the SOCS6-peptide-treated cells. In addition, we asked whether JAK2 was ubiquitinated after intracellular delivery of the SOCS6 peptide. So we performed immunoblot analysis following immunoprecipitation using anti-JAK2 antibody. The result demonstrated that the protein level of ubiquitin was markedly increased in the SOCS6-peptid-treated cells. In the non-treated cells, the bands were not detected clearly. This result indicated JAK2 bound to ubiquitin following treatment of SOCS6 peptide in epidermal stem cells. These experiments were done at least twice. These results suggested that the SOCS6 peptide-mediated neuronal differentiation of the epidermal stem cells was related to JAK2 ubiquitination followed by inhibition of the JAK2-STAT3 pathway (Figure 5A).

### 2.5. Electrophysiological Analysis of Neurological Differentiation by Use of the Patch-Cramp Configuration

We electrophysiologically analyzed SOCS6-peptide-mediated neuronal differentiation of epidermal stem cells, and recorded voltage-gated inward and outward currents in the whole-cell patch-clamp configuration [37]. In whole-cell recordings of 2 µM-SOCS6 peptide-delivered epidermal stem cells showing neurite outgrowth, the depolarizing voltage steps elicited both large outward potassium currents and fast inward Na+ currents, which are hallmark features of differentiated neurons. On the other hand, in the whole-cell recording of non-treated naïve epidermal stem cells without neurite outgrowth, neither outward potassium nor inward Na+ currents were elicited (Figure 5B).

### 2.6. Behavioral Evaluation Using the Morris Water Maze Test after Transplantation of Epidermal Stem Cells into Rodent Brain

**Figure 6:**
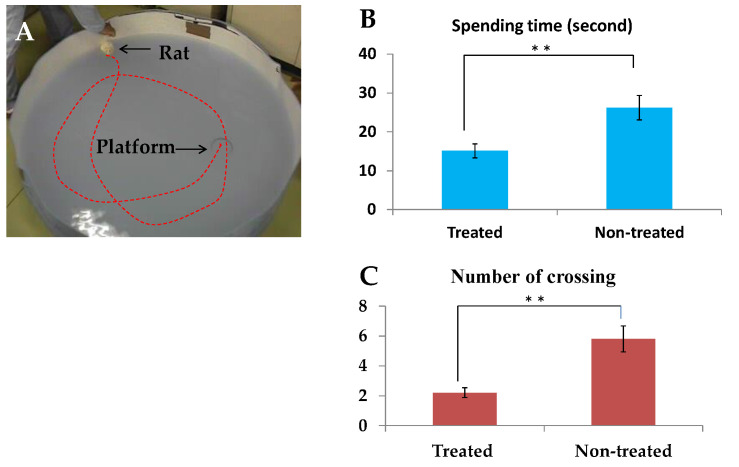
Morris water maze test. (**A**), A rat explores a platform in the pool; (**B**), Time spent to find the platform; (**C**), Number of crossings on the platform. Both spending time to find the platform and number of crossings on the platform were significantly greater in the SOCS6 group than in the non-treated group (*p* < 0.05). * *, *p* < 0.05.

Finally, we asked whether transplanted SOCS6-treated epidermal stem cells could return cognitive function to the ischemic brain; and so we performed the Morris water maze test (Figure 6A) [40]. The infarct area of a brain ischemic model rat was located in the lateral half of the left hemisphere, including the cortex, striatum, and hippocampus. The hippocampus on the lesion side was atrophic and showed a partially irregular arrangement or loss of neurons compared with the contralateral side. The mean infarct volumes in the MCAO rats on day 33 were 50.5% ± 9.6%. One week after stroke and just before transplantation, severe right-sided neurologic deficits were apparent and the rodent behaviors showed no statistical differences among the two groups. However, in the Moris water maze test, the SOCS6 group showed a shorter latency time than the non-treated-cells-transplanted control group. The mean latency time for the set on day 40 demonstrated a significant difference between the SOCS6 and control groups (*p* < 0.05). In the spatial probe trial, rats in the SOCS6 group showed improvement compared with the control group (*p* < 0.05; Figure 6). The mean time spent in the quadrant in which the platform had been located was longer in the SOCS6 group compared with the control group, and there was a statistical difference between the SOCS6 and control groups (*p* < 0.05; Figure 6).

## 3. Discussion

Epidermal stem cells are the ideal precursor cell population that can be derived in an autologous fashion from small amounts of accessible tissue biopsies and are pluripotent somatic stem cells capable of differentiating into both neural and mesodermal cells. Before cell transplantation for regeneration-aimed cell-based therapy for neuronal disease, neuronal differentiation of the donor cells is fundamental, because untreated naive cells scarcely differentiate into neurons in engrafted neural tissues. However, not only efficient generation of neurons from those stem cells but also differentiation to GABAnergic neuron phenotype from those cells has scarcely been demonstrated [41]. Therefore, an efficient neuronal differentiation method is required for differentiation to the GABAnergic neuron phenotype. Previously, we demonstrated the generation of dopaminergic neuron phenotype from neural stem cells or epidermal stem cells via the intracellular delivery of a BC-box motif peptide derived from VHL [42]. Then, in the present study we proposed a novel method using the intracellular delivery of a BC-box motif peptide derived from SOCS6 for GABAbnergic differentiation of epidermal stem cells. In this study, by confocal microscopy, we observed the rapid intracellular delivery of FITC-conjugated peptide containing the BC-box motif in SOCS6. We observed that the FITC-conjugated peptide was transferred into 77.8% ± 6.7% of the epidermal stem cells by 60 min and that these peptides were subsequently targeted to the nucleus. So far, numerous methods for neuronal differentiation of stem cells have been proposed [43,44]. In most of these methods, transcription factors are overexpressed by gene transfer [45,46]. On the other hand, we used a method of direct intracellular transfer of a synthesized peptide conjugated with a protein transduction domain, as previously reported in the literature [47,48]. Our employed method is not only safe without viral vector but also simple because only a peptide is added to the culture medium. Moreover, this method resulted in rapid transfer in 60 min as well as rapid differentiation in 24 h. For intracellular protein expression, it seems that the intracellular delivery of a cell-penetrating protein conjugated to a protein transduction domain is superior to gene transfer, which is accompanied by problems such as troublesome handling, toxicity, carcinogenicity, slowness, and infectious risk with viral vectors. However, the former approach shows less efficiency to induce differentiation to another cellular phenotype. Thus, we consider that our employed method using a protein transduction domain could be highly valuable in spite of limitations such as problems with the synthesis of peptide and protein transduction domain.

In this study using morphological, immunocytochemical, electrophysiological, and western blot methods, we observed the GABAnergic-neuronal differentiation activity of the BC-box motif peptide derived from SOCS6 protein. As for the molecular mechanism of neuronal differentiation by SOCS proteins, the molecular mechanisms of SOCS1, 2, 3, 6 involved in neuronal differentiation have been partially defined. It is known that SOCS1 and SOCS3 contain a unique KIR upstream of the central SH2 domain and that both of them inhibit JAK activity through their SH2 domain, though SOCS1 binds to JAKs while SOSC3 binds to the receptor through the SH2 domain [49,50]. Those facts suggest that forced expression of SOCS1 in NSCs promotes the generation of neurons by negative feedback inhibition of JAK2 and STAT3, as SOCS1 and SOCS3 play a negative regulatory role in signal transducer and activator of transcription 3 (STAT3). Then, it has been suggested that activities of SOCS6 to inhibit astrogliogenesis and to promote neuronal differentiation are caused by activation of STAT3 [51,52]. On the other hand, SOCS2 is a negative regulator of growth hormone signaling and regulates neurite outgrowth by regulating the phosphorylation of epidermal growth factor receptor (EFGR) [16,18]. As for SOCS6, the neuronal differentiation process by SOCS6 is suggested to be enhanced by IGF-1, which stimulates STAT5 activation, leading to up-regulation of SOCS6. In a classical negative-feedback mechanism, SOCS6 is bound to IGFR as well as JAK2, leading to inhibition of STAT5-mediated signaling. In this study, our results similarly suggest that the SOCS6-derived BC-box motif peptide leads to JAK2 ubiquitination followed by the inhibition of the JAK2-STAT3 pathway, resulting in neuronal differentiation of epidermal stem cells. Recently, we demonstrated that a BC-box motif peptide derived from VHL is concerned with JAK2 ubiquitination followed by STAT3 degradation [36]. In turn, we demonstrated that SOCS6–derived BC-box motif peptide is similarly concerned with JAK2 ubiquitination followed by STAT3 degradation.

In addition, we observed GABAnergic neuronal differentiation promoted by the SOCS6 peptide. Cell-based therapies with GABAnergic interneurons are promising for epilepsy, spasticity, Parkinson’s disease, neuropathic pain, hypoxic-ischemic injury, and Alzheimer’s disease [53]. Huntington’s disease is also a potential therapeutic target for cell therapy with GABAnergic neurons [54]. In this study, we transplanted SOCS6-peptide-delivered epidermal stem cells into the brain of cerebral infarction model rats and observed that the grafted cells differentiated into GABAnergic neurons in the rodent brain. Furthermore, these model rats showed improved cognitive function following transplantation. This result suggests that the grafted cells led to neuronal regeneration connecting neuronal networks and that transplantation of GABAnegic neurons could contribute to neuronal regenerative medicine. Briefly, we showed the differentiation of pluripotent epidermal stem cells into GABAnergic neurons, with the aim of applying this method to neuronal regenerative medicine. For further study, we might need to investigate the relationship between SOCS6-derived peptide and the GAD promoter by performing other experiments, such as chromatin immunoprecipitation, to reveal why this peptide induced GABAnergic neuronal differentiation specifically.

In conclusion, SOCS6-derived BC-box motif peptide promoted neuronal differentiation of epidermal stem cells into GABAnergic neurons. Our findings suggested that the mechanism of this peptide-mediated neuronal differentiation involved JAK2 ubiquitination followed by degradation of STAT3. When induced GABAnergic neurons were transplanted into the rat brain of a rodent ischemic model, the transplanted cells differentiated into GAD-positive cells, and these model rats showed improvement of their cognition. This study has contributed to not only elucidation of the mechanism of GABAnergic neuronal differentiation but also to neuronal regenerative medicine using GABAnergic neurons.

## 4. Experimental Section

### 4.1. Peptide Design and Synthesis

BEX. CO. LTD (Tokyo, Japan) was commissioned to do the peptide synthesis. The SOCS6-derived peptide comprises the BC box motif corresponding to the binding site of elongin C and 5 amino acids at the C-terminus of SOCS6. To facilitate the intracellular entry, we employed the protein transduction domain (PTD)-mediated peptide delivery system, by which these peptides were conjugated with PTD consisting of a modified TAT (YARAAARQARA). The amino-acid sequence of the SOCS6-derived peptide was YARAAARQARASLQYLCRFVIRQYTR (double underline, PTD; single underline, BC box motif).

### 4.2. Cell Culture and Differentiation

In this study, we used rodent skin-derived precursor cells isolated by the authors as epidermal stem cells [37]. We first examined by immunochemistry whether the cells could spontaneously differentiate into various types including neuronal cells (neuronal progenitors, astrocytes, neurons), smooth muscle cells, keratinocytes, blood cells, and fibroblasts. For morphological evaluation for neuronal differentiation, neurite outgrowth with a length exceeding the diameter of the neuronal soma was assessed as a morphological index of neuronal differentiation. In addition, for GABA neuronal differentiation, synthesized SOCS6-derived peptides at concentrations from 1 to 5 μM were added to the culture medium and delivered into the cells. Some days later, the cells were used for subsequent studies.

### 4.3. Immuncytochemistry

Immunostaining for naïve or spontaneously differentiated epidermal stem cells was performed by using anti-Neurofilament-M (NFM) (1:100; Chemicon, Temecula, CA, USA), anti-GFAP (1:200; DAKO, Santa Clara, CA, USA), anti-fibronectin (1:200; Sigma-Aldrich, St. Louis, MA, USA), anti-smooth muscle actin (SMA; 1:200; Sigma-Aldrich, St. Louis, MA, USA), anti-NGFRp75 (1:200; Sigma-Aldrich), anti-RET (1:100; LBSBio, Seattle, WA, USA), anti-keratin 15 (1:200; LBSBio), and anti-p75 NGFR (1:100; Sigma-Aldrich) antibodies as primary antibodies. In addition, immunostaining for SOCS6-induced GABAnergic cells was performed by using anti-glutamic acid decarboxylase (GAD; 1:100; Enzo Life Sciences, Farmingdale, NY, USA) and anti-gamma-aminobutyric acid (GABA; 1:200; Enzo Life Sciences) as the primary antibodies. Nuclear counterstaining was done with DAPI (Molecular Probes, Eugene, OR, USA). We counted the number of cells immuno-positive with the antibody in 6 random non-overlapping visual fields (200 cells per field). The degree of positivity was expressed as the rate of immuno-positive cells to the total number of nuclei stained with DAPI (1:5000). The following secondary antibodies were used: rhodamine-conjugated anti-IgG (1:100; Sigma-Aldrich) or FITC-conjugated anti-IgG (1:100; Sigma-Aldrich).

### 4.4. Western Blotting

Western blots were probed with anti-GAD (1:500; Enzo Life Sciences, Farmingdale, NY, USA), anti-GABA (1:500; Enzo Life Science), anti-JAK2 (1:500; Santa Cruz Biotechnology, SCB, San Diego, CA, USA), and anti-STAT3 (1:500; SCB) followed by horseradish peroxidase-conjugated secondary antibodies. Cultured cells were washed 3 times in cold PBS and then scraped into ice-cold PBS. After incubation on ice for 10 min, the cells were lysed with lysis buffer and centrifuged, after which the supernatants were collected. Each sample was separated by SDS-PAGE under reducing conditions and transferred electrophoretically to nitrocellulose filters. Non-specific binding of antibody was blocked by incubation with 5% donkey serum for 1 h. Protein bands were detected by using a chemical luminescence detection system (ECL Plus Western Blotting Reagent Pack, Amersham, Hemel Hempstead, UK). Images were analyzed with LAS-1000 (Fujifilm, Tokyo, Japan), and the density of the bands was determined by using Image Gauge software (Fujifilm).

### 4.5. Ubiqutination Assay

Total protein was extracted from the cells, and the lysates were immunoprecipitated with anti-JAK2 antibody (SCB) by using Protein A/G Sepharose. After that, each sample was separated by SDS-PAGE and transferred electrophoretically to nitrocellulose filters. Western blots were probed with anti-ubiquitin antibody (Sigma-Aldrich). Additionally, to examine the possibility of inhibition of the JAK2/STAT3 pathway, we probed Western blots with anti-JAK2 (1:500; SCB) and anti-STAT3 (1:500; SCB).

### 4.6. Electrophysiology with Patch-Cramp Configuration

To record fast sodium and delayed rectifier potassium currents, we prepared extracellular and intracellular solutions as described previously [36,40]. Five days after the addition of SOCS6 peptide at a 3-μM concentration, a holding potential of −80 mV and voltage step of 20 mV over the range of −100 to 100 mV with 50-ms durations were applied to the recorded cells through patch electrodes. For recordings and data analysis we used CEZ-2300 (Nihon Kohden, Tokyo, Japan) and pCLAMP 6.0 software (Axon Instruments, Burlingame, CA, USA). Linear components of leak and capacitive currents were reduced by analogue circuitry and then canceled by the P/N method. Signals were sampled every 20 μsec, and currents were filtered at 5 kHz. Data were additionally processed with Origin 5.0 (Microcal, Northhampton, MA, USA).

### 4.7. Middle Cerebral Artery Occlusion Rat Model

The middle cerebral artery occlusion (MCAO) procedure was modified as previously described [54]. Briefly, under deep anesthesia induced by a mixture of 1.0% to 1.5% halothane, 10% O_2_, and air, a midline cervical incision was performed; and then the left carotid bifurcation was identified. A probe made of a nylon thread with a silicon-coated head of diameter 0.3 mm was inserted into the ligated external carotid artery and advanced into the internal carotid artery to a position 16 to 18 mm from the bifurcation. During the surgery, the rectal temperature was maintained around 37.5 °C. Arterial blood gas analysis was performed, and pO_2_ was maintained at 85 to 120 mm Hg through control of the anesthetic device. Reperfusion was performed 4 h later through a 10-mm withdrawal of the probe.

### 4.8. Transplantation

On day 7 after MCAO, rats were anesthetized with an intraperitoneal injection of 50 mg/kg sodium pentobarbital and placed onto a stereotaxic frame. In a preliminary experiment, the infarct area was produced in the lateral area from approximately 3.5 mm lateral to the midline. Before grafting, cells were preincubated with red fluorescence OTH26PCL (Sigma-Aldrich). For transplantation into the non-necrotic brain parenchyma, a cell suspension, composed of 8000 to 16,000 cultured cells in 1 mL of phosphate-buffered saline (PBS, pH 7.4), was stereotaxically injected into the left forebrain from the following 3 locations: 12 mm, 0 mm, and −2 mm anterior to the bregma, and 2 mm lateral to the midline, and at a 1.2-mm depth from the cortical surface in each case. The total number of transplanted control cells was 24,000 to 48,000. Two groups of animals were prepared: (1) the control cell-transplanted group, which underwent transplantation with non-treated cells (*n* = 7); and (2) the SOCS6-treated cell-transplanted group, into which SOCS6-peptide-treated cells had been transplanted (*n* = 7).

### 4.9. Immunohistochemistry and Histology

To evaluate neuronal differentiation of transplanted cells transplanted into rat brains, we performed an immunohistochemical study as described previously [8]. The rats were perfused with periodate-lysine-paraformaldehyde solution 6 weeks after the transplantation. Their brains were subsequently dissected and postfixed in the same medium for 2 h, cryopreserved in 30% sucrose for 12 h, and then embedded in Tissue Tek OCT compound (Sakura, Tokyo, Japan). Cryostat coronal sections of 14-μm thickness were prepared for immunohistochemistry. For immunostaining, sections were incubated with primary antibody, anti-GAD (1:100; Enzo Life Sci) or anti-NeuN (1:100; Chemicon), and then with secondary antibodies, FITC-conjugated anti-rabbit IgG (1:100; Sigma-Aldrich). Counterstaining for nuclei was done with DAPI. For immunohistochemistry, observations were made with a confocal immunofluorescence microscope (FV300, Olympus, Tokyo, Japan).

### 4.10. Morris Water Maze Test

The Morris water maze test is a useful method to assess cognitive function [19]. This test was performed from day 36 to day 40 after MCAO. A pool (diameter 150 cm, depth 35 cm) was prepared with an escape platform (diameter 10 cm) located 1 cm beneath the surface of the water, which was rendered opaque and milky white. Four starting points around the edge of the pool were designated as N, E, S, and W. The platform was kept in the middle of a particular quadrant, equidistant from the center and the edge of the pool. A rat was released into the water from each starting point and allowed to swim until it reached the platform, and the time taken to reach the platform was recorded (maximum of 120 s). Rats were trained in the task using 2 sets of 4 trials on each of 5 consecutive days. After the first set on the fifth day, instead of the second set, a spatial probe trial was performed. This test is used to estimate short-term memory retention. The platform was removed and the rat was allowed to swim for 60 s. The number of times each animal crossed the area in which the platform had previously been located was measured. The time spent in the quadrant in which the platform had previously been located was also measured.

### 4.11. Ethic Approval

This study protocol was approved by the ethics committee of Yokohama City University on 15 April 2016 (YCU 20160415-4) and was in accordance with National Institutes of Health guidelines.

### 4.12. Statistics

Numerical data were presented as the mean (%) ± SEM. Factorial analysis of variance was applied to each group with pairwise comparison done by the Bonferroni method or Mann-Whitney U-test. To verify whether differences between distinct conditions reached the significance level, we used *p* < 0.05.
